# The expression of Lamin A mutant R321X leads to endoplasmic reticulum stress with aberrant Ca^2+^ handling

**DOI:** 10.1111/jcmm.12926

**Published:** 2016-07-15

**Authors:** Monica Carmosino, Andrea Gerbino, Giorgia Schena, Giuseppe Procino, Rocchina Miglionico, Cinzia Forleo, Stefano Favale, Maria Svelto

**Affiliations:** ^1^ Department of SciencesUniversity of BasilicataPotenzaItaly; ^2^ Department of Biosciences, Biotechnology and BiopharmaceuticsUniversity of BariBariItaly; ^3^ Cardiology UnitDepartment of Emergency and Organ TransplantationUniversity of BariBariItaly; ^4^ Consiglio Nazionale delle RicercheBariItaly

**Keywords:** Laminophaties, nucleus, endoplasmic reticulum, stress, apoptosis

## Abstract

Mutations in the Lamin A/C gene (*LMNA*), which encodes A‐type nuclear Lamins, represent the most frequent genetic cause of dilated cardiomyopathy (DCM). This study is focused on a *LMNA* nonsense mutation (R321X) identified in several members of an Italian family that produces a truncated protein isoform, which co‐segregates with a severe form of cardiomyopathy with poor prognosis. However, no molecular mechanisms other than nonsense mediated decay of the messenger and possible haploinsufficiency were proposed to explain DCM. Aim of this study was to gain more insights into the disease‐causing mechanisms induced by the expression of R321X at cellular level. We detected the expression of R321X by Western blotting from whole lysate of a mutation carrier heart biopsy. When expressed in HEK293 cells, GFP‐ (or mCherry)‐tagged R321X mislocalized in the endoplasmic reticulum (ER) inducing the PERK‐CHOP axis of the ER stress response. Of note, confocal microscopy showed phosphorylation of PERK in sections of the mutation carrier heart biopsy. ER mislocalization of mCherry‐R321X also induced impaired ER Ca^2+^ handling, reduced capacitative Ca^2+^ entry at the plasma membrane and abnormal nuclear Ca^2+^ dynamics. In addition, expression of R321X by itself increased the apoptosis rate. In conclusion, R321X is the first *LMNA* mutant identified to date, which mislocalizes into the ER affecting cellular homeostasis mechanisms not strictly related to nuclear functions.

## Introduction

Lamins are the only intermediate filaments localized in the nucleus, underlying the inner nuclear membrane 1. They play a crucial role in maintaining cellular as well as nuclear integrity and, together with other nuclear envelope proteins, Lamins work as a scaffold for proteins that regulate DNA synthesis, responses to DNA damage, chromatin organization, gene transcription, cell cycle progression, cell differentiation and cell migration 2, 3. Lamins have been classified into two types based on biochemical properties, expression patterns and encoding genes. B‐type Lamins are expressed in most cells and encoded by two separate genes. A‐type Lamins, known as Lamin A and C, are detected primarily in differentiated cell types and result from the alternative splicing of the Lamin A gene, *LMNA* 4.

To date, more than 450 different mutations have been identified in the *LMNA* and collected once published in the UMD‐LMNA mutation database at http://www.umd.be/LMNA/. Mutations in *LMNA* cause a group of inheritable disease phenotypes identified as Laminopathies. Most of these diseases affect specifically the striated muscle with a persistent involvement of the heart that develops dilated cardiomyopathy (DCM), conduction system disorders (CD), and arrhythmias 5. Many *LMNA* mutation carriers have a poor prognosis 6, because of a high rate of major cardiac events, such as sudden cardiac death (SD), life‐threatening ventricular arrhythmias, extreme bradycardia because of high‐degree atrio‐ventricular block and progression to end‐stage heart failure 5. In addition to DCM‐CD, some atypical forms of *LMNA*‐related cardiac diseases were reported 7, 8. Recently, severe forms of arrhythmogenic right ventricular cardiomyopathy (ARVC) have been linked to *LMNA* mutations 9 with genetic and phenotypic overlap between DCM and ARVC 9, 10, 11, 12, 13. Although the physiological role of Lamins in diverse cell functions has been precisely investigated, the molecular mechanisms induced by *LMNA* mutations and leading to the cardiac phenotypes described above are not yet fully understood 14, 15, 16.

This study is focused on a representative *LMNA* nonsense mutation that introduces a premature termination codon within the 6th of 12 *LMNA* exons producing a truncated protein isoform in the central a‐helical coiled‐coil rod domain (coil 2B) of the Lamin A protein. The resulting mutant variant of Lamin A, R321X, misses the nuclear localization signal (NLS), which is located downstream in the Lamin A protein (aa 417‐422) and co‐segregates with DCM and cardiac rhythm disturbances in affected family members 17, 18.

However, no molecular mechanisms other than Nonsense Mediated Decay of the Messenger (NMD) and haploinsufficency were proposed to explain the cardiac phenotype 17, 18. Interestingly, Geiger and collaborators showed that the efficiency of NMD seems to be tissue‐dependent since only a modest reduction of the mutant transcript was observed in the myocardium compared to skin fibroblasts, suggesting that haploinsufficiency could not be the only DCM‐causing molecular mechanism. Of note, when expressed in HeLa cells R321X has abnormal nucleoplasmic localization and a peculiar cytoplasmic distribution, with obscure impact on cell homeostasis 17.

We identify this mutation in several members of an Italian family with a frequent history of sudden death, confirming that this mutation is associated with a very severe cardiac phenotype and poor prognosis. We have been able to detect the expression of R321X both in the left and right ventricles of heart biopsies from a patient carrying this particular mutation. Thus, we tried to get more insights into the disease‐causing mechanisms first by a detailed analysis of R321X expression and localization in HEK293 cells. Interestingly, we found that R321X was not targeted to the nuclear envelope rather it accumulates in the endoplasmic reticulum (ER) and into the nucleoplasm. Functional studies showed that the presence of R321X into the ER caused the onset of the ER stress response that was in turn accompanied by ER Ca^2+^ handling abnormalities and thus increased susceptibility to apoptosis. In conclusion, this is the first Lamin A mutant identified so far which mislocalizes into the ER and affects cellular homeostasis mechanisms not strictly related to nuclear functions.

## Materials and methods

### Patients

The Lamin A mutant R321X, identified for the first time in 2006 19, has been also found in several members of an Italian family screened in our Clinical Unit dedicated to cardiomyopathies. All participants underwent clinical workup, including medical history, physical examination, 12‐lead electrocardiogram (ECG), transthoracic echocardiography and 24‐hr ECG recording. All participants provided written informed consent. This study conforms to the principles outlined in the Declaration of Helsinki and was approved by the Ethics Committee of University Hospital Consortium, Policlinico of Bari, Italy. Formalin‐fixed paraffin‐embedded heart biopsies of patients IV‐1 and a healthy control were obtained from the Hospital Niguarda Ca'Granda, Unit of Pathological Anatomy (Milan, Italy) and the Frederico II University Hospital, Unit of Pathological Anatomy (Naples, Italy) respectively.

### Generation of the Lamin A constructs

The non‐synonymous C961T nucleotide substitution was performed with Stratagene's Quik Change II XL site‐directed mutagenesis kit (Agilent Technologies, Santa Clara, CA, USA), using the PCR product of *LMNA* cDNA cloned in the pcDNA^™^6.2/N‐EmGFP‐DEST (Thermo Fisher Scientific, Waltham, MA, USA) vector system as a template 13. Mutagenic primers were designed using Quik Change Primer Design Program available online at www.agilent.com/genomics/qcpd/. The mutation was verified by sequencing. The synthetic gene mCherry (mCh) was designed with XbaI and PspXI flanking restriction sites, inserted into pMA‐RQ (ampR) and synthesized by Life technologies^™^ (Thermo Fisher Scientific). For the generation of Lamin A m‐Cherry tagged constructs, EmGFP was excised from EmGFP‐tagged Lamin A constructs and substituted with mCherry excised from pMA‐RQ vector by XbaI and PspXI (both from New England Biolabs^®^ Inc., Ipswich, MA, USA) double digestion. The final constructs were purified using QIAfilter^™^ Plasmid Maxi Kit (QIAgen, Valencia, CA, USA) and then verified by sequencing. The generation of the FLAG‐tagged R321X is described in the Supporting Information.

### Cell culture and transient transfection

HEK293 cell line was maintained in culture in DMEM high glucose, 2 mM l‐glutamine, 10% foetal bovine serum, penicillin (50 U/ml) and streptomycin (50 U/ml) at 37°C, 5% CO_2_ in a humidified incubator, and seeded for transfection. HEK293 cells were transfected with ether GFP‐(or mCh)‐Lamin A‐ or GFP‐(or mCh)‐R321X constructs using Lipofectamine 2000 reagent according to the manufacturer's instructions (Thermo Fisher Scientific). For the analysis of Ca^2+^ dynamics in the ER or in the nucleus, cells were transiently transfected either with, Cameleon D1ER 20 or nuclear Ratiometric Pericam respectively 21.

### Protein extraction and Western blotting

Protein extraction from formalin‐fixed paraffin‐embedded tissues has been performed a previously reported 22. For protein extraction from HEK293 cells transfected with Lamin A we used RIPA buffer (150 mM NaCl, 10 mM Tris HCl, 0.1% SDS, 1% Triton X‐100, 1% Na deoxycolate, 5 mM EDTA) containing phosphatase inhibitors (10 mM NaF, 1 mM sodium orthovanadate) and protease inhibitor cocktail (1:50; Roche, Basel, Switzerland) followed by sonication. Unsolubilized material was pelleted by centrifugation at 13,000 × *g* for 30 min. at 4°C.

Extracted proteins were resolved using on 4–12% precast gel (Bolt^®^BisTrisPlus gel; Thermo Fisher Scientific). Bands were electrophoretically transferred onto Immobilon‐P membranes (Merck Millipore, Billerica, MA, USA) for Western blot analysis, blocked in TBS‐Tween‐20 containing 5% BSA and incubated overnight with the following primary antibodies: anti‐Lamin A/C (dil. 1:500; Cell Signaling Technology, Inc., Danvers, MA, USA), anti‐GFP (dil. 1:500; Covance, Princeton, NJ, USA) for both mutant and wild‐type Lamin A detection, anti‐STIM1 (dil. 1:200; Santa Cruz Biotechnology, Dallas, TX, USA), anti‐SERCA2 (dil. 1:150; Santa Cruz Biotechnology, Dallas, TX, USA), anti‐ORAI (dil. 1:2000; Alomone, Jerusalem, Israel), anti‐GAPDH (dil. 1:5000; Merck Millipore), anti‐PARP, anti‐CHOP, and anti‐actin (dil. 1:500; Sigma‐Aldrich, St. Louis, MO, USA) and anti‐phospho‐PERK (dil. 1:200; Bioss Antibodies, Woburn, MA, USA). Immunoreactive bands were detected with secondary antibody conjugated to horseradish peroxidase (Tebu Bio, Le‐Perray‐en‐Yvelines, France). The following day membranes were washed and incubated with horseradish peroxidase–conjugated secondary antibody. Negative control with secondary antibody alone was performed.

### Immunofluorescence confocal analysis

For immunofluorescence confocal analysis, cells were grown on coverslips and 24 hr after transfection were fixed in PFA with 0.1% Triton X‐100 for 20 min. at RT. After washes with PBS, cells were blocked in saturation buffer [1% bovine serum albumin (BSA) in PBS] for 30 min. at room temperature (RT) and incubated with the following antibodies: anti‐Calnexin (dil. 1:500; Santa Cruz Biotechnology, Dallas, TX, USA), anti‐Nuclear Pore Complex (dil. 1:500, Covance), anti‐emerin (dil. 1:200, Santa Cruz Biotechnology, Heidelberg, Germany), anti‐SERCA2 (dil. 1:150; Santa Cruz Biotechnology, Dallas, TX, USA), anti‐FLAG M1 (dil. 1:250; Sigma‐Aldrich), for 2 hr at RT in blocking buffer. After three washes in PBS cells were incubated with 555 (or 488) Alexafluor‐conjugated secondary antibodies (Thermo Fisher Scientific) for 1 hr at RT. Nuclei were stained with either TO‐PRO‐3 (Thermo Fisher Scientific), DAPI or propidium iodide (all from Sigma‐Aldrich).

For immunohistochemical confocal analysis, serial 10 μm thin sections from formalin‐fixed paraffin‐embedded heart biopsies were attached on coverslips and deparaffinized in Histolemon (Carlo Erba, Milano, Italy) and rehydrated in a graded ethanol series. Antigen retrieval was performed by boiling sections in 50 mM glycine‐HCl (pH 3) for 30 min. Nonspecific binding sites were blocked with 1% BSA in phosphate‐buffered saline (PBS) plus SDS 0.01% for 30 min. at room temperature. Sections were then incubated with the primary antibodie (anti‐phospho PERK, 1:200; Bioss Antibodies) over night at 4°C. After washes in PBS, sections were incubated with the appropriate AlexaFluor‐conjugated secondary antibodies (Thermo Fisher Scientific) for 1 hr at room temperature. After washes in PBS, nuclei were stained with propidium iodide (Sigma‐Aldrich) and sections were finally mounted in PBS/glycerol (1:1) containing 1% n‐propylgallate, pH 8.0.

Confocal images were obtained with a confocal laser‐scanning fluorescence microscope (Leica TSC‐SP2, Mannheim, Germany).

### Evaluation of cytosolic and ER Ca^2+^ levels with either Fura‐2 or FRET‐based probe D1ER

Steady‐state‐ or real time‐FRET experiments were performed as described 23. Briefly, HEK293 cells seeded on poly‐l‐lysine‐coated glass coverslips were transiently cotransfected with plasmids encoding either mCh‐Lamin A or mCh‐R321X and the FRET‐based probe D1ER, (gift from Prof. Roger Tsien) 20. Details are available in the Supporting Information.

For intracellular Ca^2+^ measurements mCh‐Lamin A or mCh‐R321X‐HEK293 cells were loaded with 5 μM Fura‐2 (Thermo Fisher Scientific) for 25 min. at 37°C in DMEM. Coverslips with dye‐loaded cells were mounted in a perfusion chamber (FCS2 Closed Chamber System; BIOPTECHS, Butler, PA, USA) and measurements were performed with an inverted microscope (Nikon Eclipse TE2000‐S microscope) equipped for single cell fluorescence measurements and imaging analysis. Details are available in the Supporting Information.

### Apoptosis assays

Detection of apoptotic cells was performed by flow cytometry using 7‐amino‐actinomycin (7‐AAD) staining (BD Pharmingen kit, San Jose, CA, USA). In brief, HEK293 cells were seeded at a density of 10^5^ cells/well in 24‐well plates, transfected either with GFP‐Lamin A or GFP‐R321X. Forty‐eight hours post‐transfection, cells were harvested, washed twice with cold PBS, resuspended in binding buffer and incubated with 7‐AAD for 15 min. at room temperature accordingly to manufacturer's instruction. The stained cells were analysed on a FACS Canto II flow cytometer.

## Results

### Clinical and experimental data from an Italian family carrying R321X mutant

**Figure 1:**
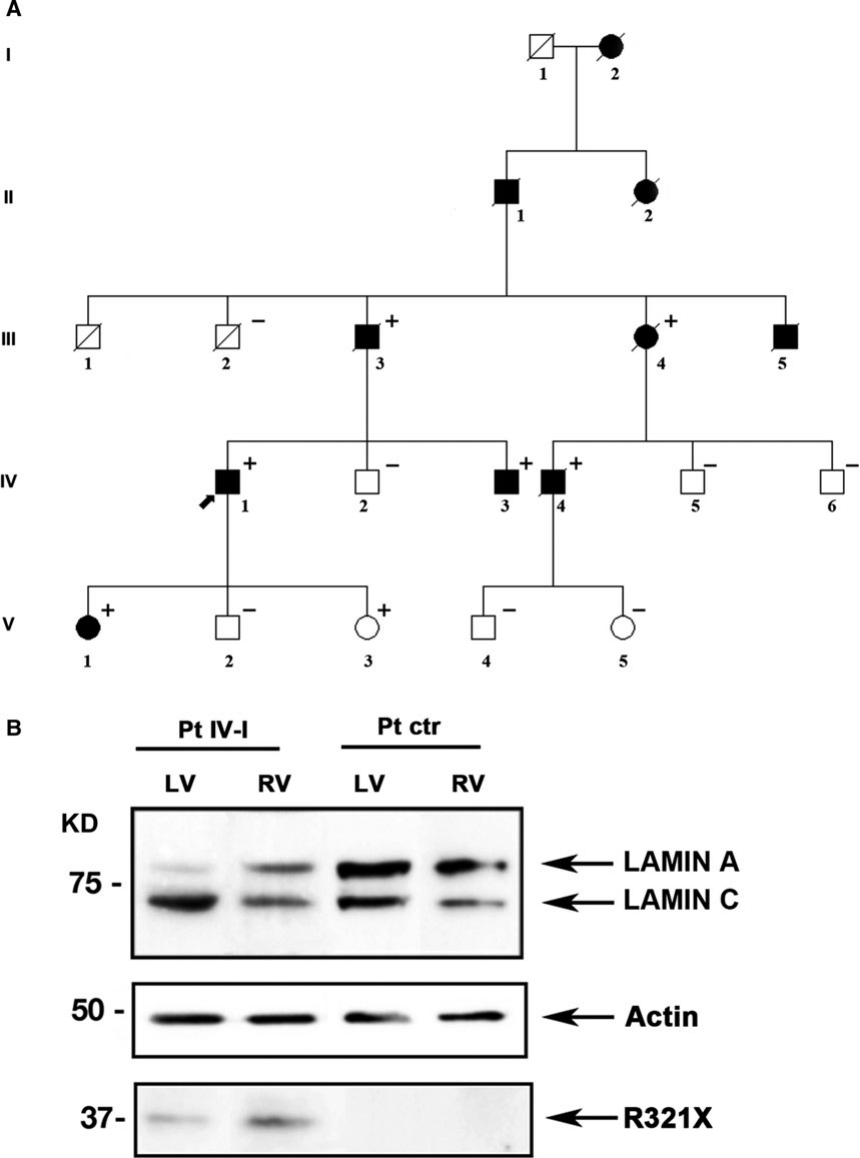
Pedigree of the family carrying the mutant R321X and its expression in the index patient (IV‐I) heart tissue. (**A**) Filled symbols indicate clinically affected individuals, diagonal slashes indicate deceased individuals; +/− indicate positive/negative for the mutation, arrow indicates the index patient. (**B**) Western blot analysis of whole lysate of left (LV) and right (RV) ventricular myocardial tissue from mutant carrier (Pt IV‐I) and control (Pt ctr) heart biopsies using a polyclonal antibody for Lamin A/C. Actin was used as loading control.

As shown in Figure 1A, the pedigree of the family was consistent with autosomal dominant transmission. The genetic screening has been performed in 14 members and 7 of them resulted positive for the mutation (+/− in the pedigree). The clinical features co‐segregating with the R321X, collected in Table 1, were consistent with different cardiac phenotypes such as arrhythmogenic cardiomyopathy, DCM, conduction disturbances, supraventricular and ventricular arrhythmias, and sudden cardiac death (SD). On the basis of family history and clinical features, it is most likely that the family members who suddenly died were carrying the same mutation indicating that the R321X induces a peculiar and severe form of cardiolaminopathy with a very poor prognosis.

**Table 1:** Clinical features of affected family members

**Patient**	**Gender**	**Age** * **(years)**	**ECG data**	**PM/ICD implantation**	**Cardiac phenotype**	**SCD/Heart transplantation**
I‐2	Female					SCD (age: 54 years)
II‐1	Male				Dilated cardiomyopathy	SCD (age: 65 years)
II‐2	Female					SCD (age: 67 years)
III‐1	Male	1†				
III‐3	Male	58	Atrial fibrillation with slow ventricular response	PM (age: 59 years)	Mildly dilated right ventricle	
III‐4	Female	58	Atrioventricular block, (age: 58 years), atrial fibrillation (age: 66 years)	PM (age: 58 years)	Dilated cardiomyopathy	
III‐5	Male	57	Atrioventricular block (age: 57 years), atrial fibrillation (age: 60 years)	PM (age: 57 years)		SCD (age: 69 years)
IV‐1	Male	32	Atrioventricular block, non‐sustained ventricular tachycardia (age: 32 years), sustained ventricular tachycardia (age: 39 years), atrial flutter (age: 44 years)	ICD (age: 39 years)	Arrhythmogenic cardiomyopathy	Heart transplantation (age: 48 years)
IV‐3	Male	50	Atrioventricular block (age: 50 years), atrial fibrillation (age: 52 years)	PM (age: 50 years)		
IV‐4	Male	43	Atrioventricular block, sick sinus syndrome (age: 43 years), sustained ventricular tachycardia (age: 48 years)	PM (age: 43 years), ICD (age: 48 years)	Dilated cardiomyopathy	
V‐1	Female	21	Non‐sustained ventricular tachycardia			

ECG, electrocardiogram; PM, pacemaker; ICD, implantable cardioverter defibrillator; SCD, sudden cardiac death; *at diagnosis/clinical presentation (years); ^†^Subject III‐1 died at the age of 1 year due to septicaemia.

Western blotting was performed to explore the expression of Lamin A and R321X in samples of left and right ventricular myocardium obtained from the index patient (IV‐1) at the time of cardiac transplantation. As control, we used cardiac muscle tissue from a patient who experienced heart transplantation due to an ischaemic heart disease not related to *LMNA* mutations (Fig. 1B).

Three different procedures for protein extraction from formalin‐fixed paraffin‐embedded heart biopsies were used 22, 24, 25. In our hands, only the protocol reported by Nirmalan *et al*. 22 enabled efficient extraction of enough immunoreactive protein to be detectable by Western blot analysis. Figure 1B shows a representative Western blotting using an antibody detecting both Lamin A and Lamin C. A protein band with an expected molecular weight of approximately 37 KD, representing the truncated mutant protein, was detected in the left and right ventricle of the R321X mutation carrier, although at a visible reduced expression levels compared to Lamin A (Pt IV‐I). This result was consistent with the transcript analyses reported by Geiger *et al*. 17, in the heart of the patient characterized in their work. On the other hand, no protein band with similar molecular weight was detectable in the cardiac muscle tissue from the control patient (Pt ctr). As expected, both the 72 KD Lamin A band and the 64 KD Lamin C band were expressed in the mutation carrier and in the control patient. The Lamin A/Lamin C protein ratio was not statistically different in the two heart samples (data not shown).

This prompted us to functionally characterize this mutation to gain more insights into disease‐causing mechanisms at cellular level.

### Expression of R321X mutant in HEK293 cells

**Figure 2:**
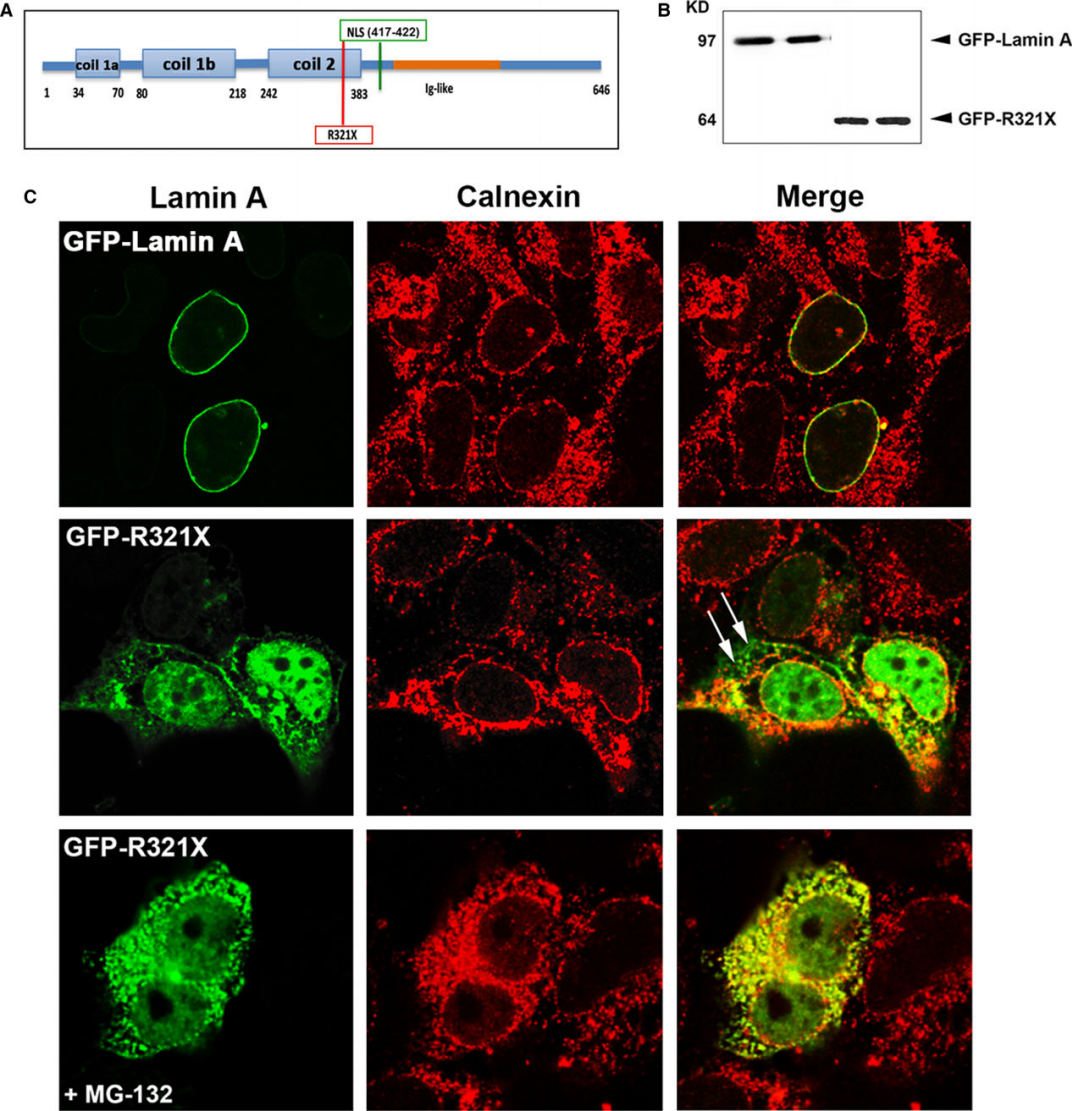
Expression and localization of GFP‐R321X in HEK293 cells. (**A**) Localization of the nonsense mutation (R321X) upstream of the nuclear localization sequence (NLS) on the Lamin A/C protein. (**B**) Lysates from HEK 293 cells expressing either GFP‐Lamin A or GFP‐R321X were subjected to Western blot analysis using a monoclonal antibody raised against GFP. (**C**) HEK293 cells were transfected with either GFP‐Lamin A (upper panel) or GFP‐ R321X (middle and lower panel) and analysed after 24 hr by confocal laser‐scanning microscopy using a polyclonal anti‐calnexin antibody (red) as ER marker. Colocalization is shown in yellow in the merge panels. Note the marked differences in Lamin A distribution in cells expressing R321X, which accumulates within the ER and the nucleoplasm compared with the normal nuclear staining in cells expressing GFP‐Lamin A. White arrows in the Merge panel indicate sites of R321X accumulation outside the ER that disappeared after pre‐treatment with the proteasome inhibitor MG‐132 (lower panel).

R321X mutant derives from a *LMNA* nonsense mutation, which introduces a premature termination codon, upstream the NLS, producing a truncated protein isoform in the coil2b of Lamin A (Fig. 2A). Here, GFP‐tagged Lamin A wild‐type (GFP‐Lamin A) and R321X (GFP‐R321X) were transiently expressed in HEK293 cells and identified by Western blotting using an anti‐GFP antibody. Figure 2B shows two immunoreactive bands of approximately 95 and 65 kD corresponding to the wild‐type (68 kD for Lamin A plus 27 kD for GFP) and the truncated protein (37 kD for R321X plus 27 kD for GFP) respectively.

Image analysis by laser‐scanning confocal microscopy showed that R321X was mainly localized in the nucleoplasm and in a cytoplasmatic vesicular compartment when compared to Lamin A, which was selectively expressed along the nuclear rim (Fig. 2B, Lamin A, R321X). Colocalization of both GFP‐Lamin A and GFP‐R321X with calnexin, a well‐known ER marker, supports the conclusion that most of R321X is localized in to the ER. As indicated by white arrows, a fraction of the R321X staining (Fig. 2C), does not overlap with calnexin staining, indicating a possible expression of R321X also in proteasomes. To test this hypothesis, we treated GFP‐R321X expressing HEK‐ cells with a well‐known proteasome inhibitor (MG‐132, 25 μM for 5 hr) and we found that the expression of GFP‐R321X was completely confined to the ER. These results likely indicate that R321X accumulated within the ER, undergoes the proteosomal degradation pathway. Of note, the expression of GFP‐R321X into the ER did not alter the localization of other nuclear envelope proteins (such as emerin and the nuclear pores, see Fig. S1), as observed for other *LMNA* mutations producing truncated version of Lamin A 26.

ER localization of R321X mutant was also evaluated in a cardiac cell line, HL‐1. As shown in Fig. S2A, when expressed in HL‐1 cells, R321X colocalized with the cardiac SERCA2 pump clearly indicating ER mislocalization of the truncated protein also in cardiac cells. In addition, ER localization of the truncated protein seems to be not related to the altered folding of the native protein induced by its fusion with GFP or mCherry tags. When R321X was tagged with a FLAG marker (of about 1 kD) at the free N‐terminus and immunolocalized with an anti‐FLAG antibody we did not find significant differences regarding R321X expression/localization (see Fig. S2B).

### The mislocalization of R321X mutant into ER induces ER stress

**Figure 3:**
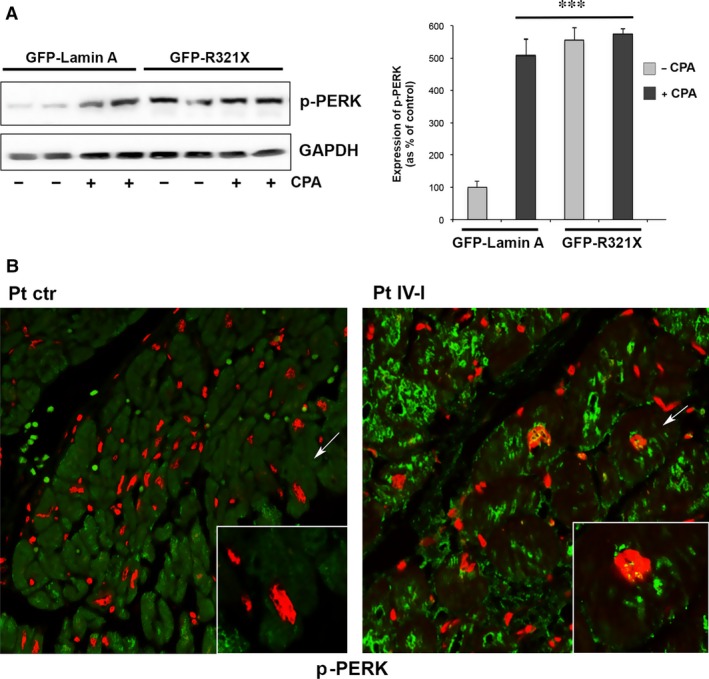
Effect of the expression of GFP‐R321X on the activation of the ER stress marker p‐PERK. (**A**) HEK 293 cells expressing either GFP‐Lamin A or GFP‐R321X were left untreated (−) or treated with the ER stress inductor CPA 100 μM for 5 hr (+). Cell lysates were subjected to Western blot analysis to detect the phosphorylation of PERK (p‐PERK). The statistical analysis graph of relative expression levels of p‐PERK was normalized to that of GAPDH. Statistical analysis was performed on three independent experiments and significance calculated by Student's *t*‐test for unpaired samples. ***P < 0.001 *versus* GFP‐Lamin A ‐CPA. (**B**) Formalin‐fixed paraffin‐embedded heart sections of the mutation carrier (right panel, Pt IV‐I) and the control patient (left panel, Pt ctr) were analysed by confocal laser‐scanning microscopy using an anti‐p‐PERK antibody (green) as ER stress marker and propidium iodide (red). Colocalization of green/red signals is showed in the merge panels.

We verified whether the inappropriate localization of a nuclear envelope protein such as Lamin A into the ER might induce the so‐called unfolded protein response (UPR). This is an adaptive mechanism involving a complex transcriptional program that induces the phosphorylation of PERK, a kinase localized on the ER membrane. PERK phosphorylates eIF2 leading to expression of transcription factors such as GADD153 (CHOP) ultimately promoting the expression of proapoptotic genes 27. We analysed the expression of phosphorylated PERK (p‐PERK) by Western blotting (Fig. 3A) in cells transfected with either GFP‐Lamin A or GFP‐R321X in control conditions or after experimental manoeuvre known to induce ER stress such as pre‐treatment with cyclopiazonic acid (CPA, 100 μM for 5 hr), a well‐known inhibitor of the sarco‐endoplasmic reticulum Ca^2+^‐ATPase (SERCA) pump 28. As shown in the densytometric analysis the expression of GFP‐R321X by itself significantly increased the expression of p‐PERK when compared with cells expressing GFP‐Lamin A (566 ± 28.4% *versus* 100 ± 20.1% *P* < 0.001). The expression of p‐PERK in GFP‐R321X expressing cells was maximal and unaffected by pre‐treatment with CPA (580 ± 12% *versus* 566 ± 28.4% *P* = n.s.), whose effect was significant only in cells expressing GFP‐Lamin A (100 ± 20.1% *versus* 509 ± 31% *P* < 0.001). These data suggest that the expression of GFP‐R321X is able, by itself, to induce phosphorylation of PERK and thus ER stress also in the absence of any further ER stress‐inducing manoeuvre. Similar results were obtained when we analysed the expression of CHOP under the same experimental conditions (Fig. S3). Next, we used scanning confocal fluorescence microscopy to evaluate the expression of p‐PERK in formalin‐fixed paraffin‐embedded heart samples obtained from both the index (IV) and control patients. Of note, a clear perinuclear staining was evident only in the cardiac cells of the index patient indicating phosphorylation of PERK in the ER and thus ER stress (Fig. 3B).

### Expression of R321X decreases ER Ca^2+^ levels by impairing both ER Ca^2+^ uptake and leak

**Figure 4:**
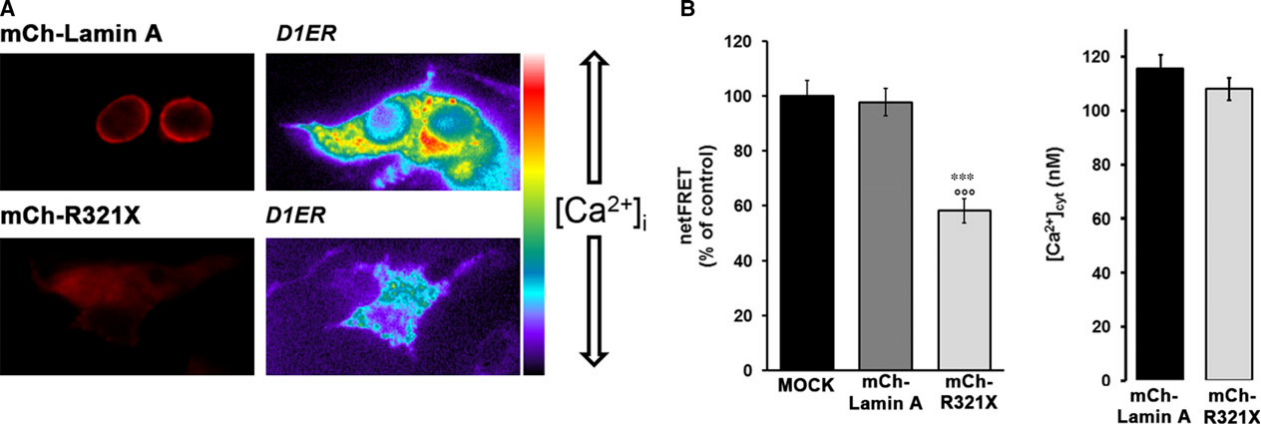
The reduction of ER Ca^2+^ levels induced by expression of R321X is not caused by changes in [Ca^2+^]_cyt_. (**A**) The steady‐state D1ER FRET signal (ratio 530/470 nm) of HEK293 cells expressing either mCh‐Lamin A or mCh‐R321X is depicted in pseudocolor. The histogram compares changes in netFRET (as % of control) among mock cells (*n* = 64 cells) and cells expressing either mCh‐Lamin A (*n* = 74 cells) or mCh‐R321X (*n* = 72 cells). Data are expressed as means ± S.E.. Statistical analysis was performed on three independent experiments and significance calculated by anova. ***P < 0.001 *versus* MOCK, °°°*P*<0.001 *versus* Lamin A. (**B**) HEK293 cells expressing either mCh‐Lamin A (black bar) or mCh‐R321X (grey bar) were loaded with Fura‐2 and the free cytosolic [Ca^2+^]_cyt_ was calculated as described in the Methods. Data are expressed as mean ± S.E.. Statistical analysis was performed on three independent experiments and significance calculated by Student's *t*‐test for unpaired samples. *P* = n.s.

**Figure 5:**
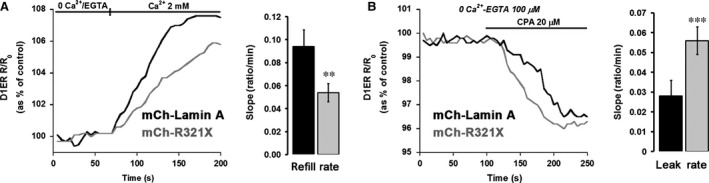
Abnormalities in ER refill and leak rate are caused by ER mislocalization of R321X mutant. (**A**) Representative traces of the speed of calcium re‐uptake into depleted ER as measured in real time with D1ER in HEK293 cells expressing either mCh‐Lamin A (black trace) or mCh‐R321X (grey trace). Statistical analysis of the refill rate based on the initial slope of the ratio increase after 2 mM Ca^2+^ readdition. Data are expressed as mean ± S.E.. Statistical analysis was performed on three independent experiments and significance calculated by Student's *t*‐test for unpaired samples. **P < 0.01 *versus* mCh‐Lamin A. (**B**) Representative traces of the speed of calcium leak from the ER after blockade of the SERCA pump as measured in real time with D1ER in HEK293 cells expressing either mCh‐Lamin A (black trace) or mCh‐R321X (grey trace). Statistical analysis of the leak rate based on the initial slope of the ratio decrease after 20 μM CPA addition. Data are expressed as mean ± S.E.. Statistical analysis was performed on three independent experiments and significance calculated by Student's *t*‐test for unpaired samples. ***P < 0.001 *versus* Lamin A.

To verify whether the observed ER stress was is turn impacting the proper functionality of the ER, we measured intraluminal ER Ca^2+^ levels with a genetically encoded FRET‐based probe, D1ER 20, in cells transfected with either m‐Cherry (mCh)‐Lamin A or mCh‐R321X. Steady‐state FRET experiments showed that ER Ca^2+^ levels measured as NetFRET were significantly reduced in cells expressing mCh‐R321X when compared with cells expressing the mCh‐Lamin A (ER Ca^2+^ levels as % of control: 58.2 ± 4.36%, *n* = 72 cells *versus* 97.7 ± 4.91%, *n* = 74 cells, *P* < 0.001) or not transfected cells (ER Ca^2+^ levels as % of control: 58.2 ± 4.36%, *n* = 72 cells *versus* 100 ± 5.6%, *n* = 64 cells, *P* < 0.001) (Fig. 4A). Since basal ER Ca^2+^ levels depends on the dynamic equilibrium between active Ca^2+^ accumulation by the SERCA pump and passive leak through calcium channels, mCh‐R321X expression could affect either process. On one hand, expression of R321X could reduce Ca^2+^ uptake by the SERCA, either by reducing the resting cytosolic [Ca^2+^] ([Ca^2+^]_cyt_) or by a direct inhibitory effect on the pump. To clarify this critical issue, we investigated these possibilities independently. At first, the possibility that mCh‐R321X affected primarily [Ca^2+^]_cyt_ (and hence ER Ca^2+^ levels only indirectly) was verified with Fura‐2. In these experiments the cells were transiently transfected with either mCh‐Lamin A or mCh‐R321X and positive cells were distinguished from controls by the typical red fluorescence emitted upon illumination with green light. As shown in Fig. 4B no difference in the resting [Ca^2+^]_cyt_ were observed between mCh‐R321X‐transfected and ‐control cells ([Ca^2+^]_cyt_: 108.08 ± 4.09 nM, *n* = 114 cells *versus* 115.54 ± 5.09 nM, *n* = 107 cells, *P* = n.s.). We then analysed whether the expression of mCh‐R321X might directly affect the activity of the SERCA pump (Fig. 5A). ER Ca^2+^ stores were first depleted with 20 μM CPA in the absence of external Ca^2+^. The rate of Ca^2+^ reuptake into the ER was measured as the initial slope of the FRET ratio increase after 2 mM Ca^2+^ re‐addition. Under these experimental conditions, the rate of Ca^2+^ entry into the ER resulted significantly lower in cells expressing R321X when compared with cells expressing the mCh‐Lamin A [Slope (ratio/min): 0.054 ± 0.008, *n* = 49 cells *versus* 0.094 ± 0.0147, *n* = 46 cells, *P* < 0.01], suggesting the functional impairment of SERCA activity in cells expressing the mutant (Fig. 5A right panel). In addition, when SERCA pump was blocked by CPA in the absence of extracellular Ca^2+^, we are able to unmask the passive ER Ca^2+^ leak (Fig. 5B). The rate of Ca^2+^ leak was measured as the initial slope of the FRET ratio decrease after CPA addition. Surprisingly, the rate of Ca^2+^ leak from the ER was significantly greater in mCh‐R321X than in mCh‐Lamin A‐expressing cells (Fig. 5B right panel), [Slope (ratio/min.): 0.056 ± 0.006, *n* = 47 cells *versus* 0.028 ± 0.007, *n* = 46 cells, *P* < 0.001], although the ER/cytosol Ca^2+^ gradient was lower in the cells expressing the mutant. Thus, we were depicting a scenario in which the expression and ER mislocalization of R321X caused generalized ER stress dramatically decreasing the Ca^2+^ levels into the ER and impairing both ER refill and leak processes. Of note, neither ER Ca^2+^‐levels nor the refill/leak rate were affected by the expression of another Lamin A mutant (DUP) expressed in the nuclear rime 13, clearly indicating that the ER mislocalization of R321X is the key event leading the cells to ER stress and abnormal Ca^2+^ handling by the ER (Fig. S4).

### ER expression of R321X affects the overall cellular Ca^2+^ dynamics

**Figure 6:**
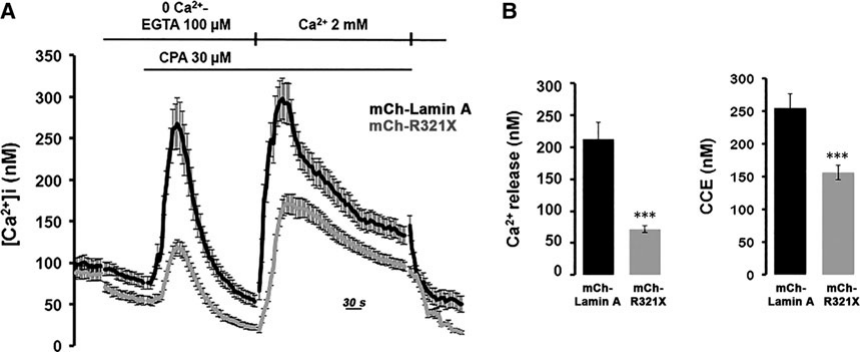
Effect of expression of R321X on both real time evaluation of ER calcium release/capacitative Ca^2+^ entry. (**A**) Time course of changes of [Ca^2+^]_cyt_ before, during, and after application of 20 μM CPA in the absence (0 Ca^2+^) or presence of extracellular Ca^2+^ (2.0 mM) in Fura‐2 loaded HEK293 cells expressing either mCh‐Lamin A (black trace) or mCh‐R321X (grey trace). (**B**) Summarized data of the amplitude of CPA‐induced Ca^2+^ release in the absence of extracellular Ca^2+^, and amplitude of CPA‐induced capacitative Ca^2+^ entry. mCh‐Lamin A, black bars; mCh‐R321X, grey bars. Data are expressed as mean ± S.E.. Statistical analysis was performed on three independent experiments and significance calculated by Student's *t*‐test for unpaired samples. ***P < 0.001 *versus* mCh‐Lamin A.

**Figure 7:**
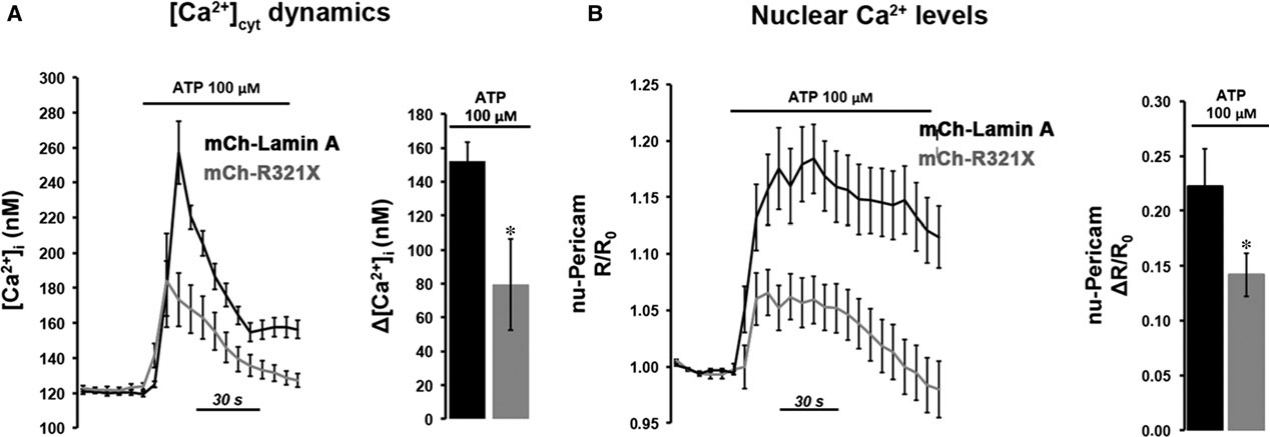
Real time evaluation of ATP‐induced cytosolic and nuclear Ca^2+^ changes. (**A**) Fura‐2 loaded HEK293 cells expressing either mCh‐Lamin A or mCh‐R321X were challenged with the Ca^2+^‐mediated agonist ATP. Histogram showing the ATP‐induced Δ[Ca^2+^]_cyt_ in mCh‐Lamin A (black bar) and mCh‐R321X (grey bar) HEK293 cells. Data are expressed as mean ± S.E.. Statistical analysis was performed on three independent experiments and significance calculated by Student's *t*‐test for unpaired samples. *P < 0.05 *versus* mCh‐Lamin A. (**B**) nu‐Pericam‐HEK293 cells expressing either mCh‐Lamin A or mCh‐R321X were challenged with the Ca^2+^‐mediated agonist ATP. Histogram shows the ATP‐induced ΔR/R0 in mCh‐Lamin A (black bar) and mCh‐R321X (grey bar) HEK293 cells. Data are expressed as mean ± S.E.. Statistical analysis was performed on three independent experiments and significance calculated by Student's *t*‐test for unpaired samples. *P < 0.05 *versus* mCh‐Lamin A.

It was previously reported that a reduction of ~30% in steady‐state ER Ca^2+^ levels results in a substantial activation (>50%) of the so‐called capacitative Ca^2+^ entry (CCE) 29. The reduction in steady‐state ER Ca^2+^ levels caused by R321X expression is thus expected to cause an activation of this pathway. Therefore, we measured CCE in mCh‐R321X‐ and mCh‐Lamin A‐transfected cells loaded with Fura‐2 AM (Fig. 6A). The cells were treated with the SERCA blocker (20 μM CPA) while perfused with Ca^2+^‐free medium. This procedure first evoked a transient increase in [Ca^2+^]_cyt_ due to release of Ca^2+^ from the stores. As expected by the lower ER Ca^2+^ levels in mCh‐R321X‐expressing cells (Fig. 4B), CPA‐induced ER Ca^2+^ release was significantly reduced when compared with cells expressing the mCh‐Lamin A (Fig. 6B, Ca^2+^ release, Δ[Ca^2+^]_cyt_: 71.63 ± 5.08 nM, *n* = 57 *versus* 212.72 ± 5.08, *n* = 60, *P* < 0.001). When the release of stored Ca^2+^ was complete, 2 mM Ca^2+^ was re‐added to the medium. This manoeuvre evoked a second, larger [Ca^2+^]_cyt_ increase *via* CCE, which was markedly lower in mCh‐R321X–transfected cells (Fig. 6B, CCE, Δ[Ca^2+^]_cyt_: 155.71 ± 10.69 nM, *n* = 57 *versus* 254.29 ± 21.83, *n* = 60, *P* < 0.001). This evidence indicates that, together with the mechanisms identified so far, the specific expression of R321X into the ER is also able to impinge a mechanism important to the Ca^2+^ influx from the extracellular space. Interestingly, the expression levels of the proteins involved in the Ca^2+^ handling were not significantly different under GFP‐R321X expression when compared with GFP‐Lamin A‐expressing cells (Fig. S5), suggesting that the ER mislocalization of R321X is the key event leading the cells to ER stress and abnormal Ca^2+^ handling. Of note, the impaired ER Ca^2+^ mobilization observed in mCh‐R321X‐expressing cells was also mirrored into the nucleoplasm. We elicited an IP3R‐mediated Ca^2+^ release form the ER using ATP, a physiological agonist of the purynergic receptors endogenously expressed in HEK293 cells 30 and we monitored either cytoplasmic or nucleoplasmic Ca^2+^ dynamics using Fura‐2AM and nuclear ratiometric Pericam (nu‐Pericam) 21 respectively. The increases in [Ca^2+^]_cyt_ and in nucleoplasmic Ca^2+^ levels were both significantly lower in cells expressing mCh‐R321X when compared to mCh‐Lamin A‐expressing cells (Fig. 7A, right panel, Δ[Ca2+]_cyt_: 79.54 ± 27.06 nM, *n* = 56 *versus* 152.23 ± 11.01 nM, *n* = 48, *P* < 0.05; Fig. 7B, right panel, nu‐Pericam ΔR/R_0_: 0.142 ± 0.02, *n* = 30 *versus* 0.222 ± 0.034, *n* = 28, *P* < 0.05). Given the continuity between the ER and the nuclear envelope, it may also possible that the impairment of ER Ca^2+^ homeostasis results from a general weakening of the nuclear envelope induced by the expression of R321X. We ruled out this possibility by measuring nuclear Ca^2+^ permeability that resulted unaffected under expression of mCh‐R321X (Fig. S6). Therefore, the impairment of Ca^2+^ handling in the nucleoplasm might be of extreme physiological importance since its potential effect on genes regulation.

### Expression of R321X increases the rate of cell apoptosis

**Figure 8:**
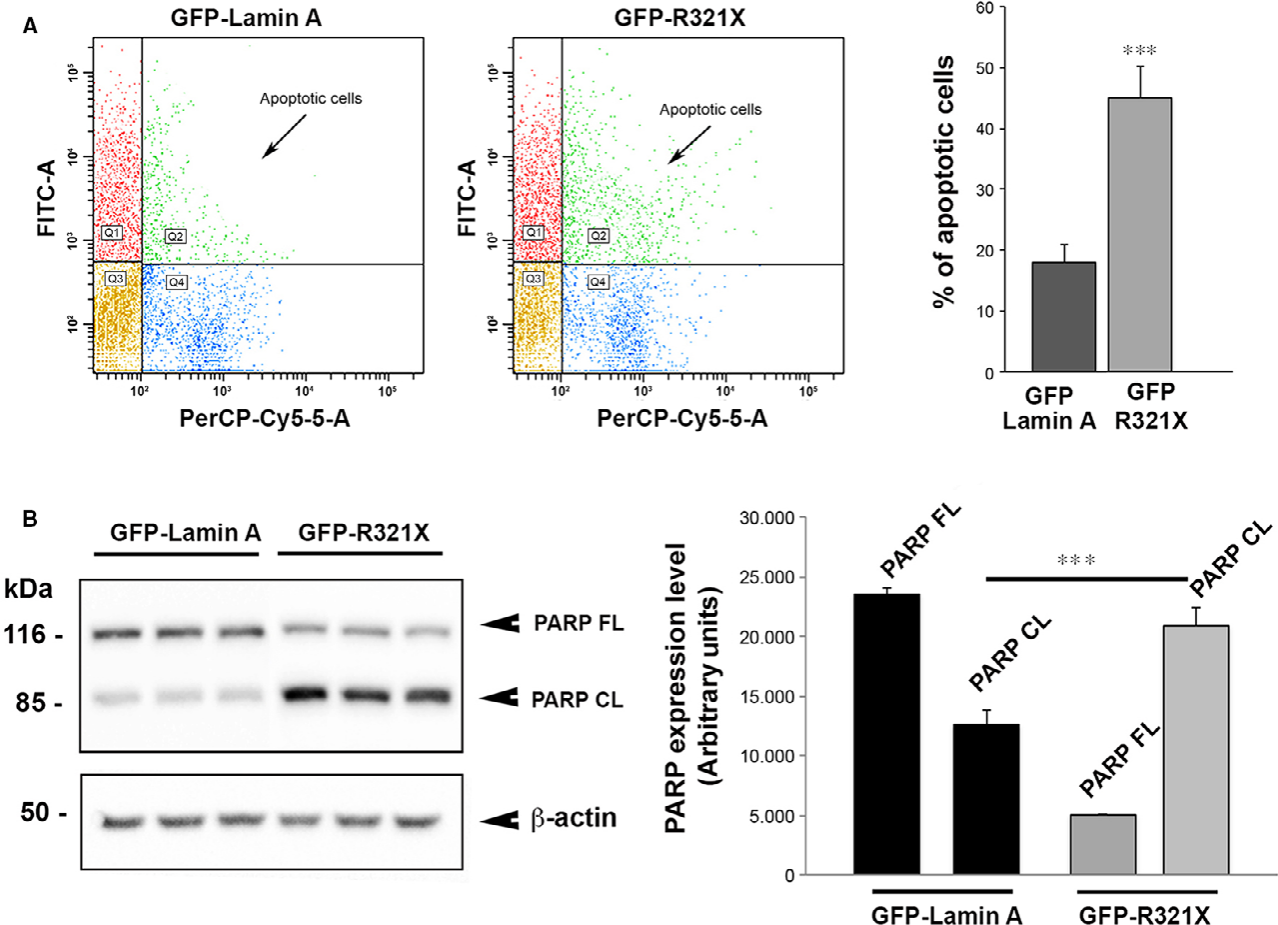
LMNA R321X expression by itself increases the rate of cell apoptosis. (**A**) Detection of apoptosis by flow cytometry. Expression of GFP‐R321X increased the % of apoptotic cells when compared with cells expressing GFP‐Lamin A. Data are expressed as mean ± S.E.. Statistical analysis was performed on three independent experiments and significance calculated by Student's *t*‐test for unpaired samples. ***P < 0.001. PerCP‐CY5.5‐A, peridinin‐chlorophyll proteins conjugate with CY5.5, FITC, fluorescein isothiocyanate, Q1, GFP positive cells; Q2, GFP positive apoptotic cells; Q3, GFP negative cells; Q4, GFP negative apoptotic cells. (**B**) Lysates from HEK293 cells expressing either GFP‐Lamin A or GFP‐R321X were subjected to Western blot analysis to detect the expression of either full length‐ or cleaved PARP (PARP FL and PARP CL respectively). The statistical analysis graph of relative expression levels of PARP FL and PARP CL normalized to that of β‐actin. Each experiment was repeated in triplicate. Data are expressed as mean ± S.E.. Statistical analysis was performed on three independent experiments and significance calculated by Student's *t*‐test for unpaired samples. ***P < 0.001 *versus* GFP‐Lamin A –PARP FL.

The events described so far may in turn increase the ER stress condition finally inducing cell death by apoptosis. To verify this hypothesis we measured the rate of cell apoptosis in either GFP‐Lamin A or GFP‐R321X expressing cells by cytofluorimetry. As shown in Figure 8A, Lamin A and R321X‐expressing cells were stained with 7‐AAD to assess the apoptotic cell populations. We showed that the apoptotic rate significantly increased up to 45% in GFP‐R321X transfected cells compared to control cells (Fig. 8A, right panel, % of apoptotic cells: 45 ± 5.2% *versus* 18 ± 3.2%, *P* < 0.001). One of the most common signalling cascades involved in apoptosis is the activation of a highly specialized family of cysteinyl‐aspartate proteases (caspases). Once activated, caspases initiate cell death by cleaving and activating effectors such as the Poly (ADP‐ribose) polymerase (PARP) 31. Once cleaved, an 85 kD fragment was excited from the 116 kD intact PARP molecule. As shown in Figure 8B, in GFP‐R321X‐expressing cells the product of caspases‐dependent PARP cleavage (85 kD band) was clearly overexpressed compared to the full length un‐cleaved PARP molecule (PARP expression level, arbitrary units: 20,876 ± 1565 *versus* 5000 ± 40.46, *P* < 0.001), confirming a significantly increase in apoptosis rate in these cells compared to GFP‐Lamin A‐expressing cells (PARP expression level, arbitrary units: 20,876 ± 1565 *versus* 12,588 ± 1256, *P* < 0.001).

## Discussion

It has been extensively reported that mutations in *LMNA* are able to mislocalize either Lamin A/C or well‐known Lamin A/C‐binding partners 13, 16, 17, 32. However, *LMNA* mutations described so far mislocalized only within the nuclear compartment, thus compromising both nuclear envelope stiffness/resistance and gene profile expression. On the basis of this observation, two opposing ‘structural’ and ‘gene expression’ models have been postulated to explain how mutations within *LMNA* gene give rise to distinct diseases. Loss of Lamin A/C function, for example, may result in nuclear fragility leading to the death of individual cells upon mechanical stress or other environmental factors. Alternatively, abnormal Lamin A/C function could alter tissue/cell type‐specific gene expression either through impaired interaction between Lamin A/C and transcription factors/regulators or through changes in the spatial organization of chromatin 15.

R321X mutant derives from a *LMNA* nonsense mutation that introduce a premature termination codon within the 6th of 12 *LMNA* exons before the NLS producing a truncated protein isoform in the central a‐helical coiled‐coil rod domain of Lamin A (Fig. 2A).

In patients carrying the same mutation, the messenger coding the mutated version of R321X was largely degraded by NMD in skin fibroblasts 17, 18 and to a lesser extent in the heart 17, 18. Accordingly, the truncated R321X protein was not easily detectable in patient tissues, suggesting the *LMNA* haploinsufficency as the only pathomechanism underlying the cardiomyopathy observed in the mutant carriers. However, only a modest reduction of the mutant transcript was observed in the myocardium of these carriers compared to skin fibroblasts 17 clearly suggesting that the NMD may not be efficient enough to avoid the harmful effects of the expressed R321X in affected tissues, especially in the heart, which is constantly under intense mechanical stress and thus prone to disorders by even weak cellular imbalance.

Moreover, several findings from meta‐analysis of previous literature, suggest that in humans severe early phenotypes are associated with dominant negative or toxic gain of function mechanisms because of the presence of mutated Lamin A. In contrast, late onset and/or milder phenotypes may arise through loss of function secondary to haploinsufficiency 33. In addition, the mild cardiac phenotype of heterozygous *LMNA* ^*+/−*^ knock‐out mice confirms that the Lamin A haploinsufficiency *per se* cannot account for severe phenotypes co‐segregating with several *LMNA* mutations 34.

We found that the expression of R321X is associated, in an Italian family, with several cases of SD at early age and severe cardiomyopathies, clearly suggesting the dominant negative effect of this Lamin A mutation.

Of note, we have been able to detect by Western blotting the truncated mutant protein in the left and right ventricle of the R321X mutation carrier (Pt IV‐I) at the expected molecular weight, although at a visible reduced expression levels compared to the wild‐type Lamin A. This result was consistent with the transcript analyses reported by Geiger *et al*. 17 in the heart of the patient characterized in their work.

As expected, both the 72 KD Lamin A band and the 64 KD Lamin C band were also expressed in the mutation carrier. A skewed ratio of Lamin A/Lamin C proteins expression has been reported in fibroblasts from skin biopsies of DCM patients carrying the same mutation 18. Although we found the same trend in heart of patient IV‐I, we noticed that this result was highly dependent on both the region of the heart analysed (left *versus* right ventricles) and on the experimental variability. Therefore, the involvement of skewed Lamin A to lamin C ratio in the onset of DCM needs further validation, especially in the cardiac tissue.

We then functionally characterized this Lamin A mutant and we demonstrated that R321X profoundly affects the ER functions when expressed in cells. We found that this mutant accumulates into ER, consistent with a previous study in which an *in vitro* Lamin A construct lacking the NLS sequence was found to accumulate into the ER compartment 35. In addition, we found that R321X accumulates into the nucleoplasm *via* either a passive diffusion process of the protein through the nuclear pores or by a ‘piggybacking’‐mediated mechanism, in other words, by a preassembled complex with a protein containing a NLS 36.

**Figure 9:**
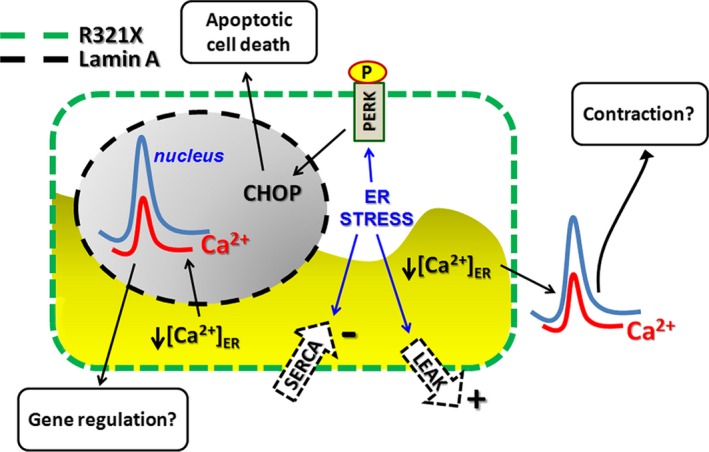
Schematic diagram illustrating the effects induced by expression of R321X in HEK293 cells. ER mis‐localization of R321X elicits the ER stress response leading to: (*i*) activation of the PERK‐CHOP signalling pathway leading to apoptosis; (*ii*) ER abnormal Ca^2+^ handling leading to impaired cytosolic and nuclear Ca^2+^ signalling which in turn might impact Ca^2+^‐mediated process such as gene regulation and contraction in cardiac cells.

However, a pool of A‐type Lamins within the nucleoplasm, which is distinct from nuclear envelope peripheral Lamin A, has been already identified in both physiological 37 and pathological 38 conditions. Thus, the ER localization of this LMNA mutant represents the novel feature of this Lamin A mutated variant. We depicted a scenario in which the introduction of the premature stop codon in the *LMNA* gene, before the NLS nuclear localization signature, produces a truncated and unfolded version of Lamin A, which likely is unable to leave the ER and to continue its biosynthetic pathway. The accumulation of R321X into the ER impairs the ability of this organelle to properly handle Ca^2+^. Finally, we found that the overall cellular response to both misfolded Lamin A accumulation into the ER and luminal Ca^2+^ depletion is to increase cell apoptosis rate, *via* activation of cellular caspases and the PERK‐CHOP cascade, involved in transcriptional responses to cellular stress. We dissected all the ER mechanisms involved in the luminal and cytosolic Ca^2+^ homeostasis in both Lamin A and R321X‐expressing cells. Notably, ER severely lost the ability to both release and re‐uptake Ca^2+^ as well as to functionally couple to the plasma membrane to allow the CCE from the extracellular space in R321X‐expressing cells. We found that the ER stress induced by R321X impaired CCE, SERCA activity and ER Ca^2+^ leak. Lack of proper Ca^2+^ homeostasis caused both failure in restoring normal ER Ca^2+^ levels and impaired agonist‐induced Ca^2+^ changes both in the cytosol and in the nuclear compartment. Interestingly, neither the expression of SERCA nor the expression of STIM1 and ORAI1 resulted affected in R321X‐expressing cells, rather suggesting a functional impairment of these proteins. We can speculate that defective STIM1‐ORAI1 coupling occurs because of the loss of ER physiological morphology and redistribution of STIM1 to ER sheets. Accordingly, a direct correlation between ER morphology and Store Operated Calcium Entry (SOCE) functionality has been recently reported 39. Although the increase in Ca^2+^ leak from ER has been demonstrated during ER stress 40, the decrease in SERCA activity has never been reported under this condition. It is possible, however, that a decrease in ATP production in mitochondria, secondary to a decrease in ER Ca^2+^ dynamics 41, 42, may impair the SERCA activity in R321X expressing cells. Depending on the duration and severity of the ER stress, cells can re‐establish normal ER function or lead to cell death by apoptosis. We found that the expression of R321X significantly increased the rate of cell apoptosis in resting state, suggesting a severe and un‐compensated ER stress in R321X‐expressing cells. It is known that ER Ca^2+^ store depletion by itself can induce ER stress and cell apoptosis 43, 44. It is indeed possible that the loss of luminal Ca^2+^ caused by ER stress further reinforces the ER stress response leading to cell apoptosis. Moreover, the impairment in Ca^2+^ dynamics into the nucleus may shift the transcription profile towards pro‐apoptotic genes 45. Downstream consequences of impaired ER functions are expected and reported in cardiomyopathy. Disruption of ER homeostasis has been linked to several processes of cardiovascular diseases including ischaemia/reperfusion injury 46, DCM 47 and HF 48, often associated to ryanodine receptors mutations 49. The disruption of ER homeostasis in cardiomyocytes has, as expected, consequence on the impairment of cardiac contractility. The consequent cell apoptosis may in turn account for conduction defects, arrhythmia and sudden death as previously discussed 13. A schematic drawing summarizing our working hypothesis is shown in Figure 9.

Advances in the design of novel compounds and therapeutic strategies to manipulate levels of ER stress in disease have been recently reported 50 and most importantly some pharmacological agents used in clinical settings such as pravastatin, pioglitazone, Tempol were reported to affect UPR pathways and ER disfunction 51, suggesting novel therapeutic strategies for this severe form of cardiac laminopathy induced by R321X mutant expression.

In addition, we have shown that R321X likely undergoes degradation *via* proteasome‐mediated mechanism just like another abnormal Lamin A protein termed progerin 52. Gabriel *et al*. showed that treatment with the activating proteasomal activity, sulforaphane, enhanced the degradation of progerin, decreased the formation of insoluble progerin aggregates and induced clearance through proteasomal mechanisms in affected and normal fibroblasts. This evidence might provide a potential therapeutic strategy also for other mutants of Lamin A, such as R321X.

## Conflict of Interest Statement

The authors confirm that there are no conflicts of interest.

## Supporting information


**Figure S1** Effect of GFP‐R321X expression on the localization of other nuclear envelope proteins analysed by immunofluorescence confocal microscopy.
**Figure S2** Expression of GFP‐R321X and FLAG‐R321X in HL‐1 cardiomyocytes and HEK293 cells respectively.
**Figure S3** Effect of the expression of GFP‐R321X on the induction of the ER stress marker CHOP.
**Figure S4** Localization and ER Ca^2+^ dynamics in HEK293 cells expressing GFP‐DUP and mCh‐DUP respectively.
**Figure S5** Expression analysis of the players involved in the Ca^2+^ cellular dynamics.
**Figure S6** Evaluation of nuclear Ca2+ permeability in mCh‐Lamin A and mCh‐R321X transfected HEK293 cells expressing the nuRatiometric Pericam.
