# Supplementary material for: The expression of Lamin A mutant R321X leads to endoplasmic reticulum stress with aberrant Ca2+ handling
Source: J Cell Mol Med. 2016 Jul 15;20(11):2194–207. doi: 10.1111/jcmm.12926 (PMC5082401; doi:10.1111/jcmm.12926)
Supplement: Supplementary file 1 — Figure S1 Effect of GFP‐R321X expression on the localization of other nuclear envelope proteins analysed by immunofluorescence confocal microscopy. Figure S2 Expression of GFP‐R321X and FLAG‐R321X in HL‐1 cardiomyocytes and HEK293 cells respectively. Figure S3 Effect of the expression of GFP‐R321X on the induction of the ER stress marker CHOP. Figure S4 Localization and ER Ca2+ dynamics in HEK293 cells expressing GFP‐DUP and mCh‐DUP respectively. Figure S5 Expression analysis of the players involved in the Ca2+ cellular dynamics. Figure S6 Evaluation of nuclear Ca2+ permeability in mCh‐Lamin A and mCh‐R321X transfected HEK293 cells expressing the nuRatiometric Pericam. [file JCMM-20-2194-s001.docx]

**Supplementary Material and Methods**

**Protein extraction from formalin fixed paraffin embedded heart tissues.**

Serial 10µm thin sections from formalin fixed paraffin embedded heart biopsies were deparaffinized in Histolemon (Carlo Erba, Milano, Italy), rehydrated in a graded ethanol series and centrifuged to remove excess of ethanol. Samples were then solubilized in freshly prepared Laemmli buffer (100 mM Tris-HCl, pH=6.8, 2% w/v SDS, 20% v/v glycerol, 4% v/v β-mercaptoethanol) heated at 99°C for 20 min. Samples were cooled for 5 min on ice, sonicated and then centrifuged (14000xg for 15 min) for supernatant collection.

**Generation of FLAG-tagged R321X.**

FLAG-tagged R321X was generated by PCR using GFP-Lamin A as a template, forward primer 5’- AAAA GTTAACA ATGGAGACCCC GTCCCAG -3’ and a reverse primer containing a stop codon 5’-AAAA GAATTC TCA AAGCTTCGCCTCCTTGGCT. PCR fragment was then digested with HpaI and EcoRI and cloned into pcDNA3 FLAG vector (ThermoFisher Scientific, Waltham, MA, USA). The construct was verified by sequencing (Eurofins MWG Operon, Ebersberg, DE).

**Evaluation of ER Ca^2+^ levels with the FRET-based probe D1ER**

Steady state- or real time-FRET experiments were carried out using MetaMorph software or Metafluor software (both from Molecular Devices, MDS Analytical Technologies, Toronto, Canada), respectively. ECFP and citrine were excited at 435 or 509 nm, respectively. FRET from ECFP to citrine was determined by excitation of ECFP and measurement of fluorescence emitted from citrine. For steady state FRET experiments FRET values were calculated as NetFRET according to the formula: NetFRET signal = FRET signal – a x YFP signal – b x CFP signal, where a and b are the ratio of the signal in FRET channel to the signal in YFP channel in the absence of donor and to the signal in CFP channel in the absence of acceptor respectively. For statistical analysis, GraphPad Prism software (version 5.00, GraphPad Software, San Diego, California, USA) was used. Significant differences between means were tested by one-way analysis of variance (ANOVA) with Newman-Keuls’s post-test. Significance was accepted for p values < 0.05.

**Evaluation of cytosolic and nuclear Ca^2+^ levels**

For intracellular and nuclear Ca^2+^ measurements, cells were seeded on poly-L-lysine-coated glass coverslips (Ø 40 mm). Ringer’s Solution was used to perfuse cells during the experiment containing 140 mM NaCl, 5 mM KCl, 1 mM MgCl2, 10 mM Hepes, 5 mM Glucose, 1.0 mM CaCl_2_, pH 7.4. HEK293 cells were stimulated with a variety of drugs as described in the results, including adenosine triphosphate (ATP), cyclopiazonic acid (CPA), digitonin and ionomycin (all from Sigma-Aldrich, St. Louis, USA). mCh-Lamin A or mCh-R321X-HEK293 cells were loaded with 5 µM Fura-2 for 25 min at 37°C in DMEM. Coverslips with dye-loaded cells were mounted in a perfusion chamber (FCS2 Closed Chamber System, BIOPTECHS, Butler, U.S.A.) and measurements were performed using an inverted microscope (Nikon Eclipse TE2000-S microscope) equipped for single cell fluorescence measurements and imaging analysis. The sample was illuminated through a 40X oil immersion objective (NA = 1.30). The Fura-2 loaded sample was excited alternately at 340 and 380 nm every 5 seconds. Emitted fluorescence was passed through a dichroic mirror, filtered at 510 nm (Omega Optical, Brattleboro, VT, USA) and captured by a cooled CCD camera (CoolSNAP HQ, Photometrics, Tucson, AZ, USA). Fluorescence measurements were performed using Metafluor software (Molecular Devices, MDS Analytical Technologies, Toronto, Canada). For determination of intracellular Ca^2+^ concentration images were corrected for the background fluorescence and calibrated as described by Grynkiewicz [1] according the following equation: [Ca^2+^]_i_ = Kd*Q(R-Rmin)/(Rmax-R), where Kd (224 nM) indicated the dissociation constant of Fura-2AM for Ca^2+^_i_ and Q indicated the ratio of the fluorescence intensities (F) at the minimum and the maximum Ca2+ concentration at 380 nm. Each sample was calibrated by the addition of 5 µM ionomycin in presence of 0.5 mM EGTA (Rmin) followed by 5 µM ionomycin in 10 mM CaCl_2_ (Rmax). For nuclear calcium measurements HEK293 cells were transiently co-transfected with plasmids encoding either mCh-Lamin A or mCh- R321X and the nuclear Ratiometric Pericam (nu-Pericam, gift from Prof. A.Miyawaki) [2]. The nu-Pericam sample was excited alternately at 485/20 and 420/30 nm every 5 seconds. Emitted fluorescence was passed through a dichroic mirror, filtered at 510 nm (Omega Optical, Brattleboro, VT, USA). The experiments were performed using the imaging set-up and the perfusion apparatus described above. The fluorescence ratio was recorded and calculated as the change in fluorescence normalized to the basal fluorescence ratio observed in the absence of stimulus (R/R_0_).

Data are expressed as means ± SE. Statistical analysis was performed on at least 3 independent experiments and significance calculated by Student**’**s T-test for unpaired samples. Significance was accepted for p values < 0.05.

**Supplementary Figures**


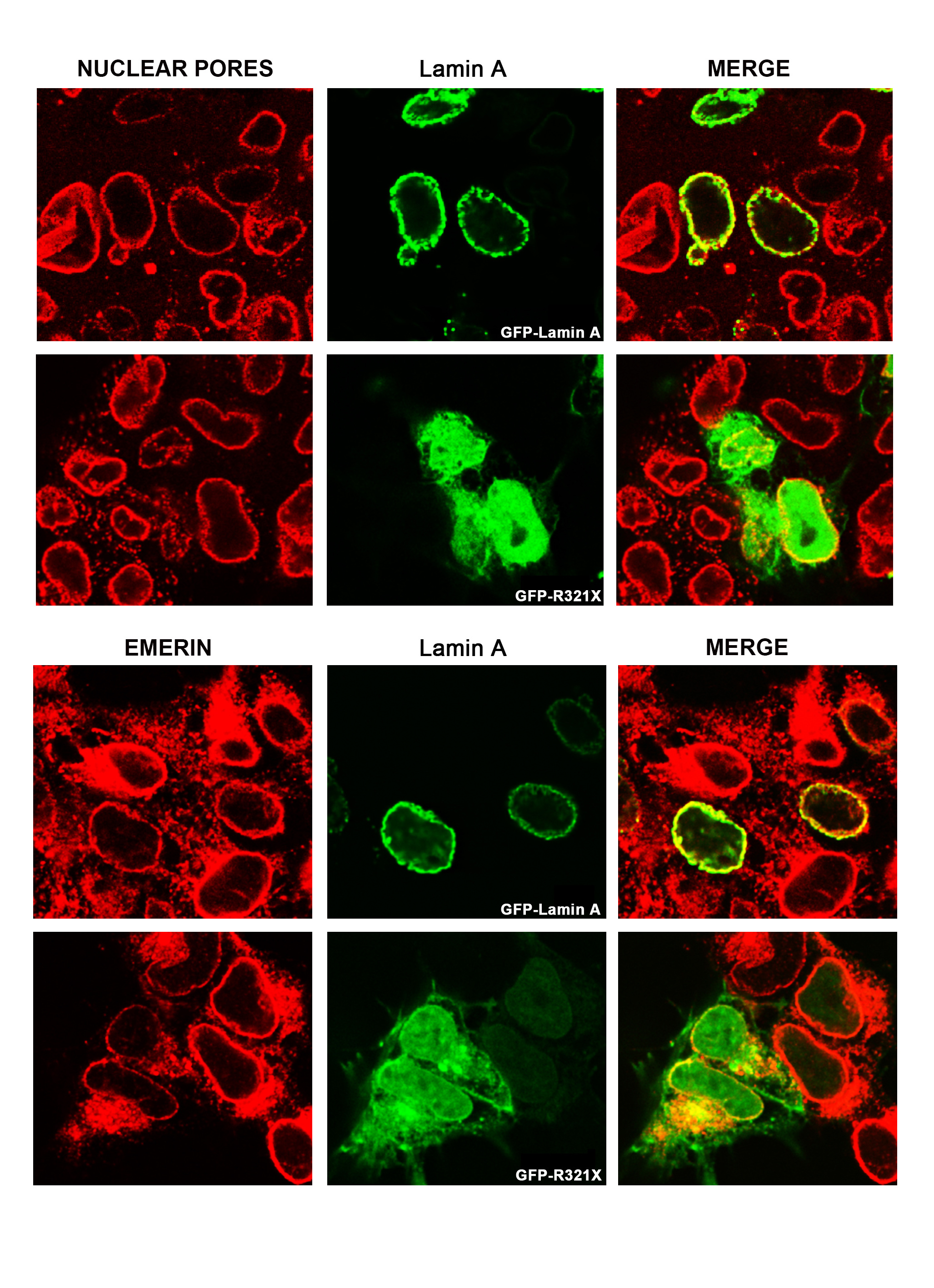


**Supplemental Fig. 1: Effect of GFP-R321X expression on the localization of other nuclear envelope proteins analysed by immunofluorescence confocal microscopy.**

HEK293 cells were transfected with either GFP-Lamin A or GFP-R321X and analysed after 24 h by confocal laser scanning microscopy using an anti-nuclear pore complex antibody (upper panels, NUCLEAR PORES, red) or an anti-emerin antybody (lower panels, EMERIN, red). Colocalization is shown in yellow in the merge panels.


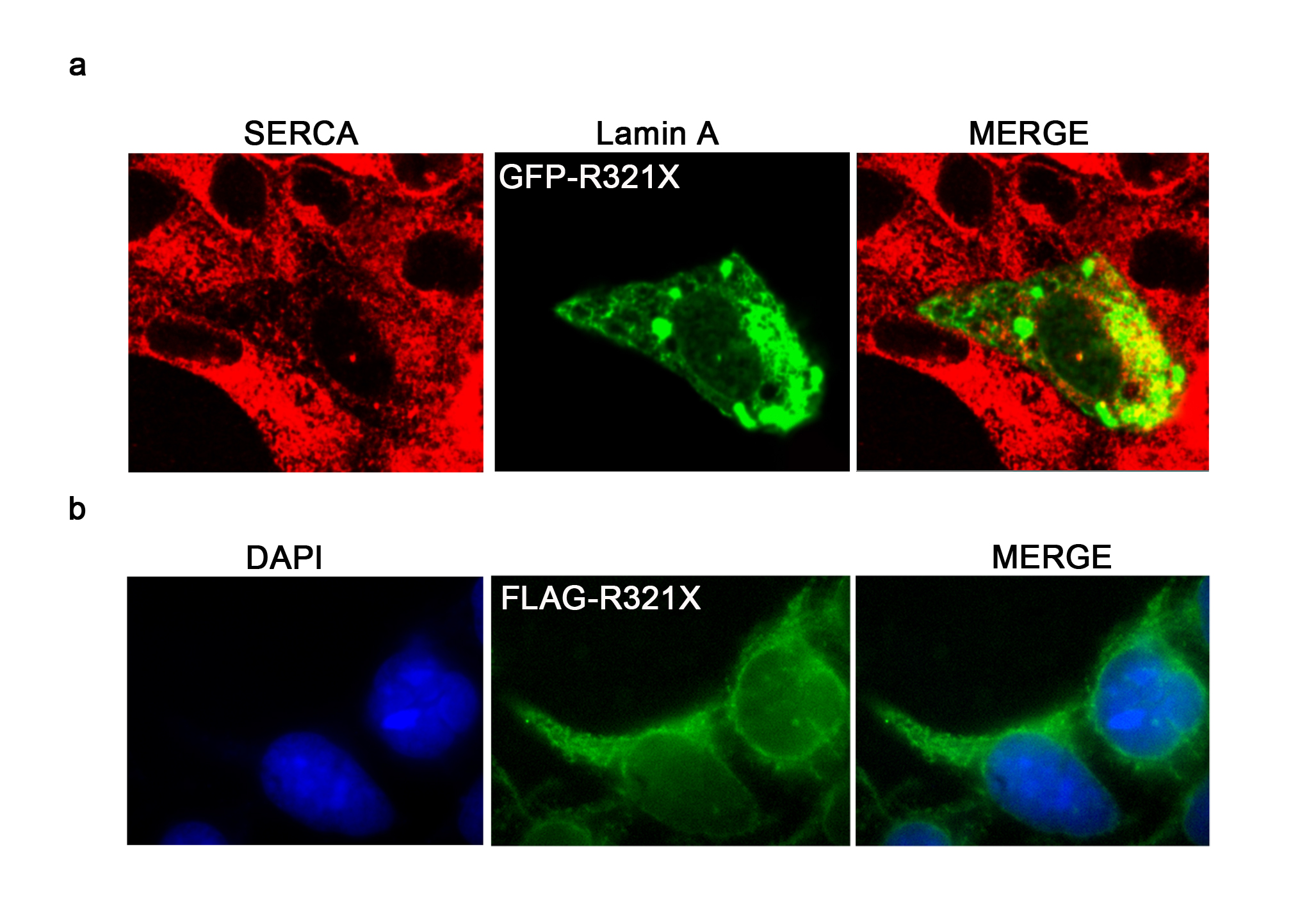


**Supplemental Fig.2: Expression of GFP-R321X and FLAG-R321X in HL-1 cardiomyocytes and HEK293 cells, respectively.** a) TransIT® Transfection Reagent (Mirus Bio LLC, Madison, WI, USA) was used to transfect HL-1 cardiomyocytes with GFP-R321X. After 24 h cells were analysed by confocal laser scanning microscopy using an anti-SERCA2 antibody (left panel, SERCA, red). Colocalization is shown in yellow in the merge panels. b) HEK293 cells were transfected with FLAG-R321X and analysed after 24 h by confocal laser scanning microscopy using an anti-FLAG M1 monoclonal antibody. Colocalization with the nuclear marker DAPI is shown in the merge panels.


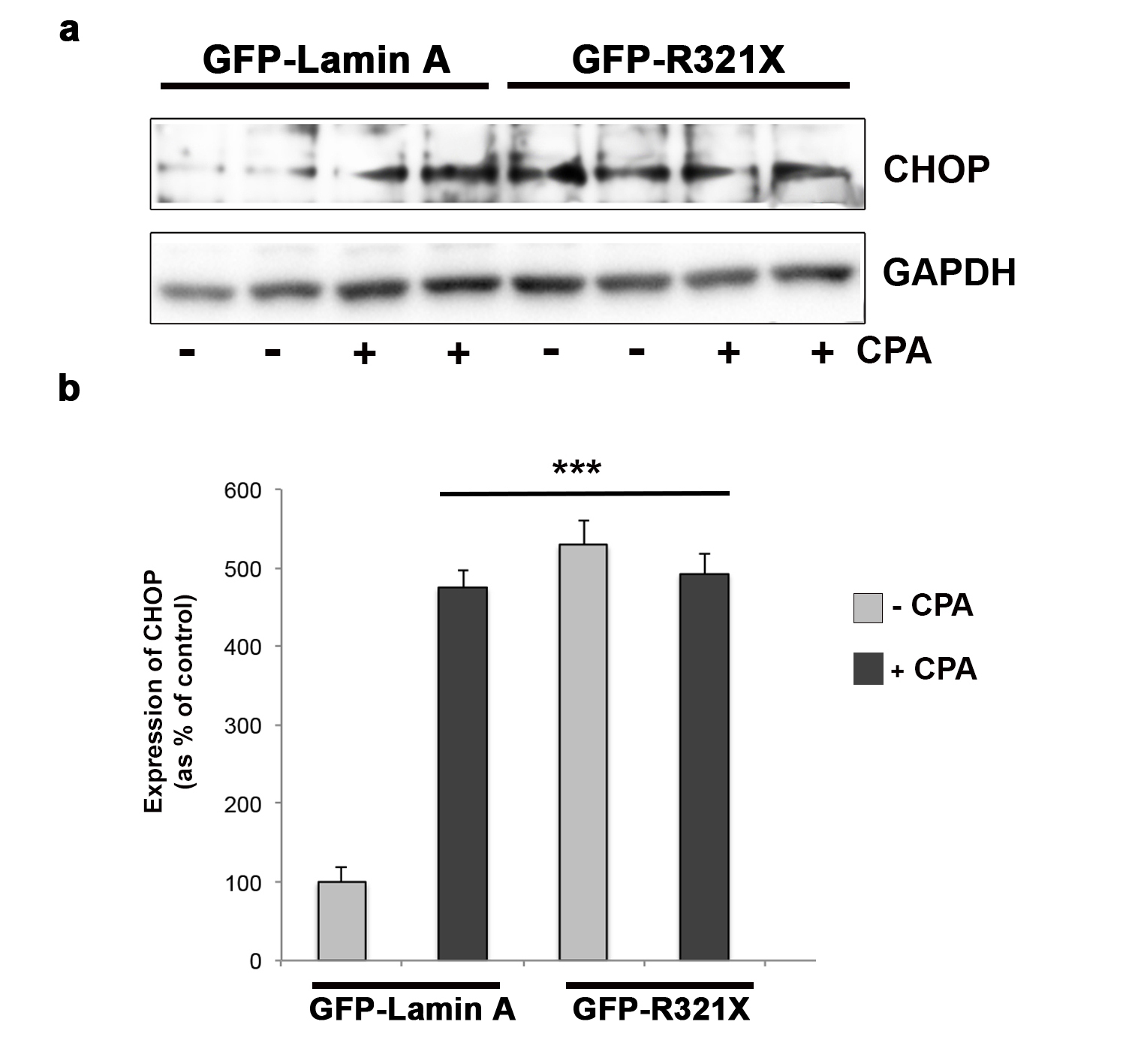


**Supplemental Fig. 3: Effect of the expression of GFP-R321X on the induction of the ER stress marker CHOP.** a) HEK 293 cells expressing either GFP-Lamin A or GFP-R321X were left untreated (-) or treated with the ER stress inductor CPA 100 µM for 5 hours (+). Cell lysates were subjected to Western blot analysis to detect the induction of the transcription factors CHOP. b) The statistical analysis graph of relative expression levels of CHOP was normalized to that of GAPDH. Expression of GFP-R321X by itself significantly increased the expression of CHOP when compared with cells expressing GFP-Lamin A (529% ± 31.4 vs 100% ± 18.7 p<0.001). The expression of CHOP in GFP-R321X expressing cells was maximal and unaffected by pretreatment with CPA (529% ± 31.4 vs 492% ± 25.7 p=n.s.), whose effect was significant only in cells expressing GFP-Lamin A (100% ± 18.7 vs 472% ± 21.2 p<0.001). Statistical analysis was performed on 3 independent experiments and significance calculated by Student’s T-test for unpaired samples. ***P < 0.001 vs GFP-Lamin A -CPA.


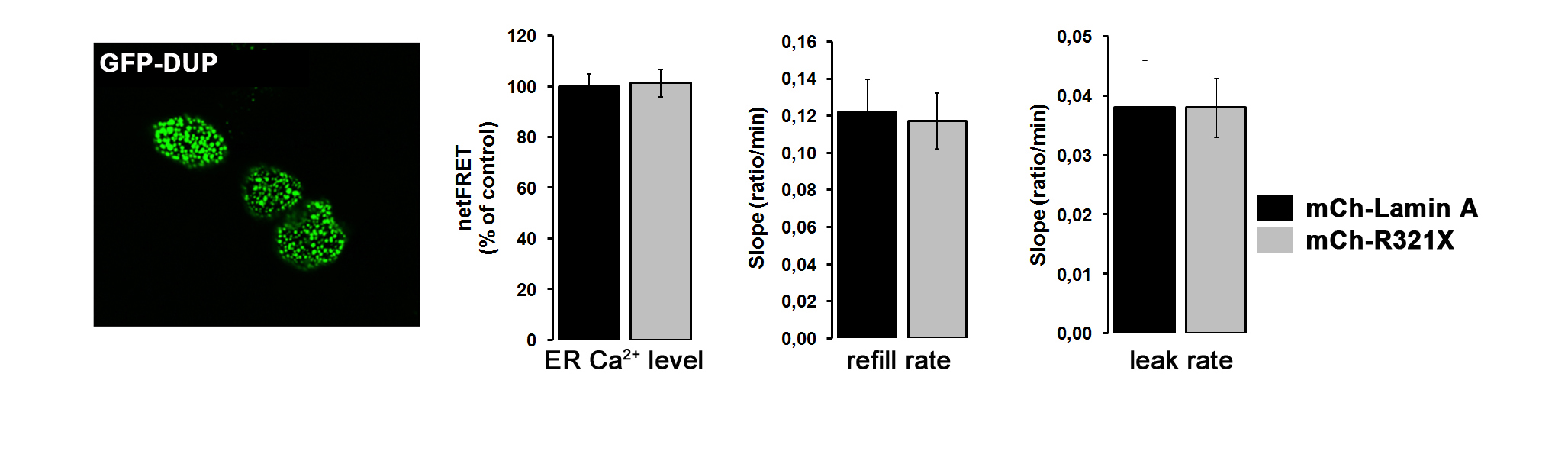


**Supplemental Fig. 4: Localization and ER Ca^2+^ dynamics in HEK293 cells expressing GFP-DUP and mCh-DUP, respectively.**

HEK293 cells transfected with GFP-DUP (left panel) were analysed after 24 h by confocal laser scanning microscopy. Note the abnormal spotted nuclear localization of the GFP-DUP that, however, does not diffuse outside the nuclear envelope if compared with GFP-R321X. Statistical analysis of the ER Ca^2+^ level, refill and leak rate in mCh-DUP cells did not show any significant difference when compared with cell expressing mCh-Lamin A (right panel). Data are expressed as means ± SE. In steady-state and real-time FRET-experiments significant differences between means were tested by one-way analysis of variance (ANOVA) with Newman-Keuls’s post-test. Statistical analysis was performed on at least 3 independent experiments. P=n.s.


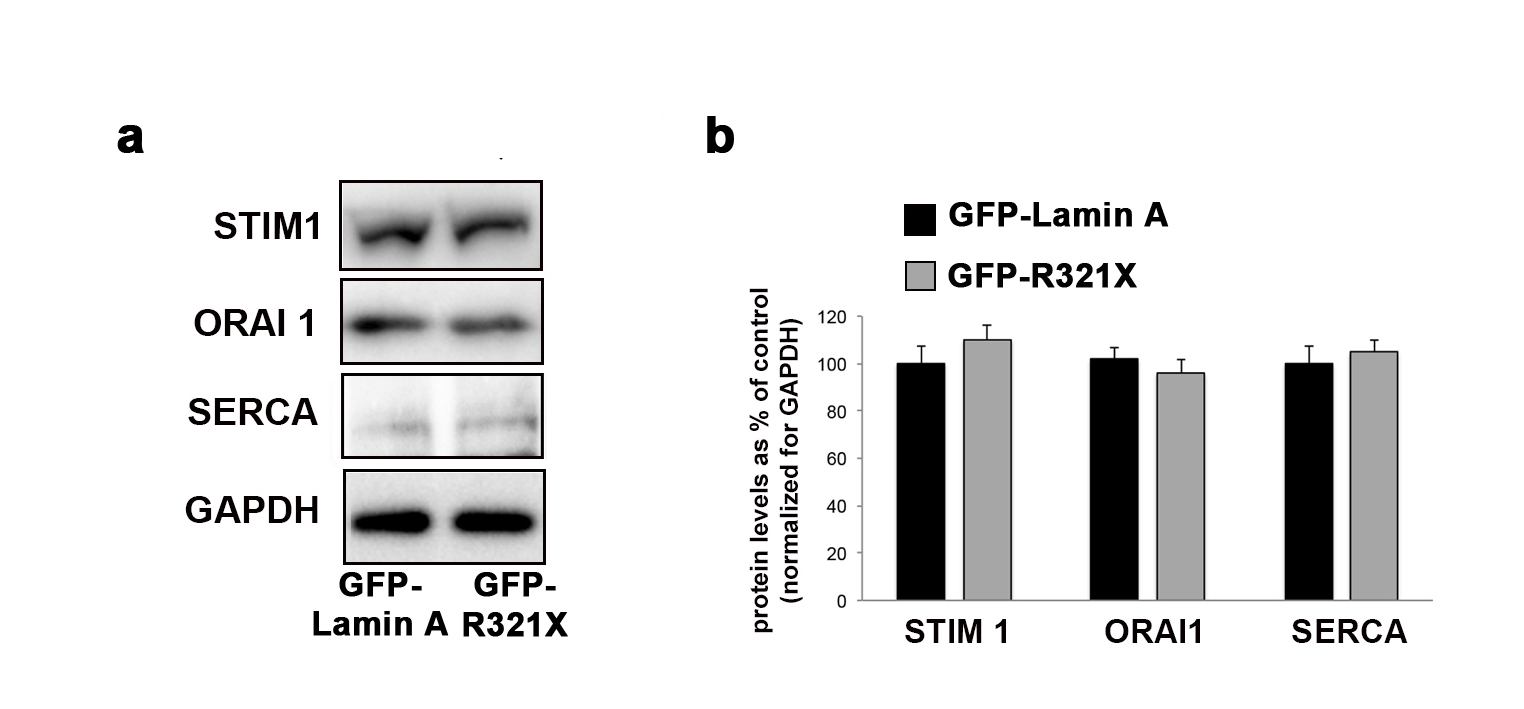


**Supplemental Fig. 5: Expression analysis of the players involved in the Ca^2+^ cellular dynamics**

a) HEK293 cells transfected with both GFP-Lamin A and GFP- R321X were lysed and analyzed for the expression of SERCA2, STIM1 and ORAI1 by western blotting. b) Statistical analysis on SERCA2, STIM1 and ORAI1 expression levels did not show any significant difference between GFP-R321X and GFP-Lamin A expressing cells. Thus, the ER inability to properly handle Ca^2+^ found upon GFP-R321X expression, was not related to the impaired expression of three major players involved in Ca^2+^ homeostasis, rather suggesting their functional impairment in R321X-expressing cells.

**
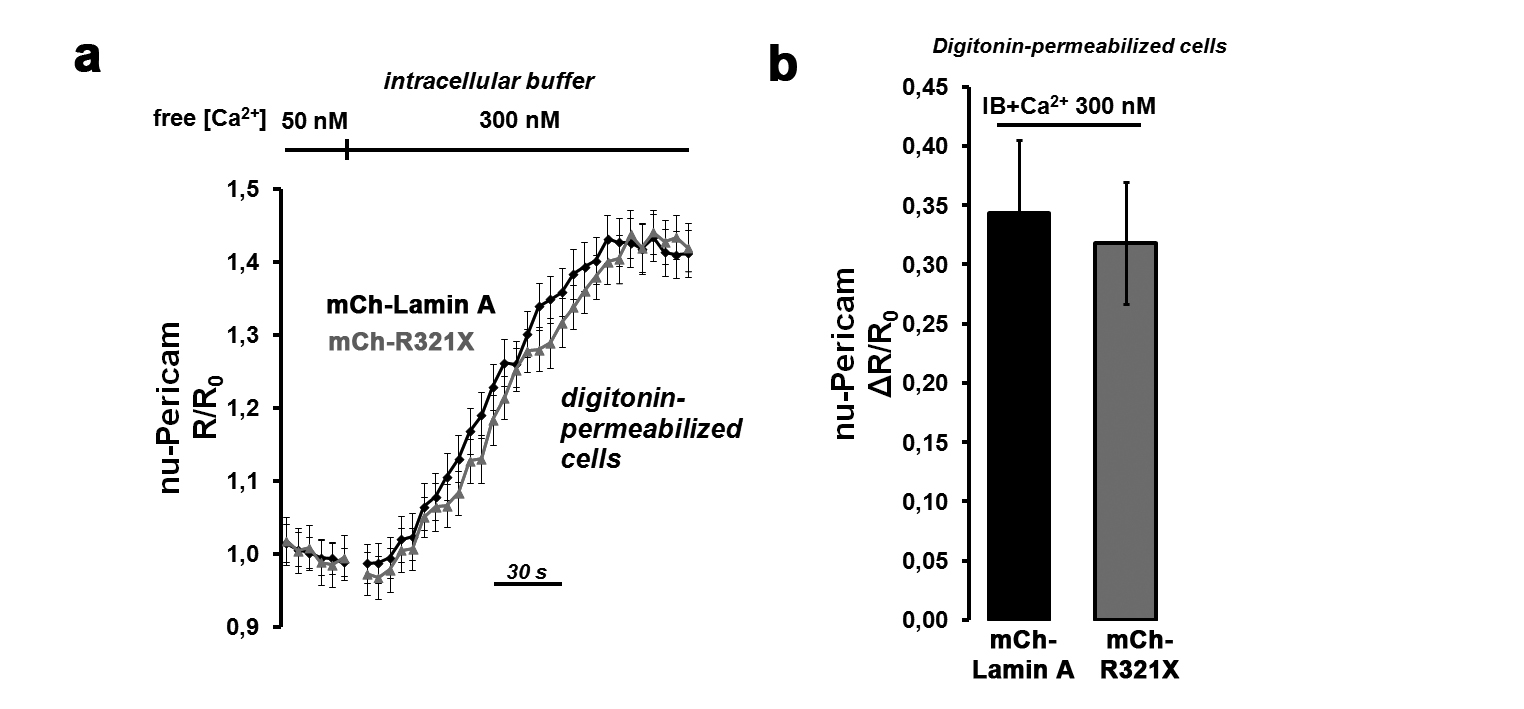
**

**Supplemental Fig. 6: Evaluation of nuclear Ca^2+^ permeability in mCh-Lamin A and mCh-R321X transfected HEK293 cells expressing the nuRatiometric Pericam.**

a) mCh-Lamin A and mCh-R321X transfected HEK293 cells expressing the nuRatiometric Pericam were rinsed briefly in a high K^+^ solution (in mM: 125 KCl, 25 NaCl, 10 HEPES, pH 7.25, 0.1 MgCl_2_), then exposed for at least 5 min to an intracellular buffer (IB) at 37°C (the same solution supplemented with 0.5 mM MgATP, pH 7.25 and Ca^2+^/EGTA buffers, 1mM total [EGTA], 50 nM free Ca^2+^, calculated according to the software maxchelator (http://maxchelator.stanford.edu/) also containing 5 µg/ml digitonin. This approach did not affect ER membrane structure or activity as proved by many reports where it was used to record ER intraluminal Ca^2+^ dynamics with the Ca^2+^ low affinity dye Mag-Fura-2 [3-5]. In addition, preliminary experiments were performed to strictly control the extent of the digitonin-induced permeabilization by measuring in real time the loss of the cytosolic Ca^2+^ dye Fura-2. After plasma membrane permeabilization, cells were perfused continuously with an intracellular buffer (without digitonin) containing 300 nM free Ca^2+^, an intracellular [Ca^2+^] recorded under maximal stimulation with 100 μM ATP (see figure 7a). a) The rapid nuclear Ca^2+^ increase induced by the perfusion with IB+300 nM free Ca^2+^ in mCh-R321X-expressing cells was not significantly different when compared with cells expressing mCh-Lamin A. b) Summarized data of the amplitude of the nuclear Ca^2+^ increases recorded in permeabilized cells when the IB free [Ca^2+^] was increased from 50 to 300 nM. mCh-Lamin A, black bars; mCh-R321X, gray bars. Data are expressed as means ± SE. Statistical analysis was performed on at least 3 independent experiments and significance calculated by Student**’**s T-test for unpaired samples. These results likely indicate that expression of R321X did not impair nuclear Ca^2+^ permeability.

**Bibliography**

[1] G. Grynkiewicz, M. Poenie, and R. Y. Tsien, "A new generation of Ca2+ indicators with greatly improved fluorescence properties," *J Biol Chem,* vol. 260, pp. 3440-50, Mar 25 1985.

[2] T. Nagai, A. Sawano, E. S. Park, and A. Miyawaki, "Circularly permuted green fluorescent proteins engineered to sense Ca2+," *Proc Natl Acad Sci U S A,* vol. 98, pp. 3197-202, Mar 13 2001.

[3] A. M. Hofer and T. E. Machen, "Technique for in situ measurement of calcium in intracellular inositol 1,4,5-trisphosphate-sensitive stores using the fluorescent indicator mag-fura-2," *Proc Natl Acad Sci U S A,* vol. 90, pp. 2598-602, Apr 1993.

[4] A. M. Hofer, W. R. Schlue, S. Curci, and T. E. Machen, "Spatial distribution and quantitation of free luminal [Ca] within the InsP3-sensitive internal store of individual BHK-21 cells: ion dependence of InsP3-induced Ca release and reloading," *FASEB J,* vol. 9, pp. 788-98, Jun 1995.

[5] A. M. Hofer, S. Curci, T. E. Machen, and I. Schulz, "ATP regulates calcium leak from agonist-sensitive internal calcium stores," *FASEB J,* vol. 10, pp. 302-8, Feb 1996.
